# Effects of Volume Overload: A Case Report of an Edema Bulla

**DOI:** 10.5070/M5.52206

**Published:** 2026-01-31

**Authors:** Jarom Morris, Matthew Sommer, Felix Braun, Brent Klapthor, Allison Beaulieu, Megan Fix

**Affiliations:** * University of Utah, Department of Emergency Medicine, Salt Lake City, UT

## Abstract

We present a case of a 75-year-old female with a history of congestive heart failure who developed a large edema bulla on her right shin after running out of her home medications. The patient presented with swelling and a rapidly enlarging bulla that reached the size of a grapefruit. Physical examination revealed bilateral pitting edema and a 10 x 10 cm bulla filled with serous fluid. Dermatology was consulted, confirming the diagnosis of edema bulla secondary to acute volume overload. The bulla was drained in the emergency department, and the patient was discharged with wound care instructions, including the application of petroleum jelly and vinegar compresses. Follow-up three months later showed significant healing. This case highlights the importance of recognizing edema bullae in patients and managing the underlying fluid overload. This case report specifically demonstrates that edema bullae can present at sizes larger than the 1–5 cm range described in existing literature.

**Topics:**

Bulla, vesicle, dermatology, blister.

**Figure:**
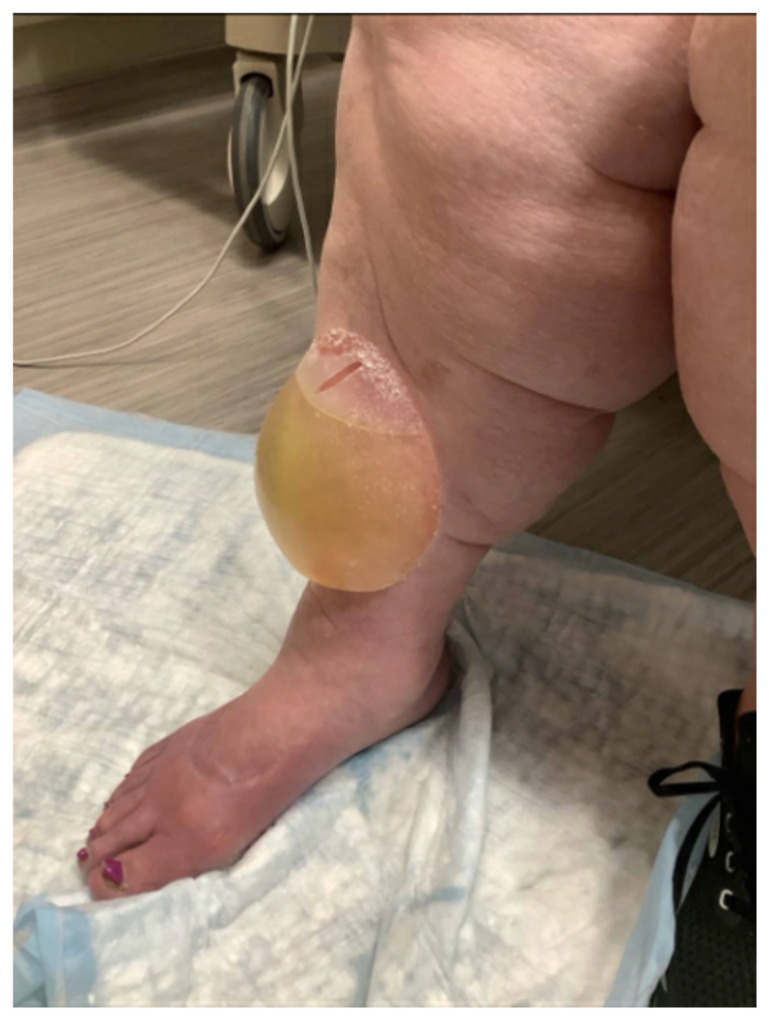


**Figure:**
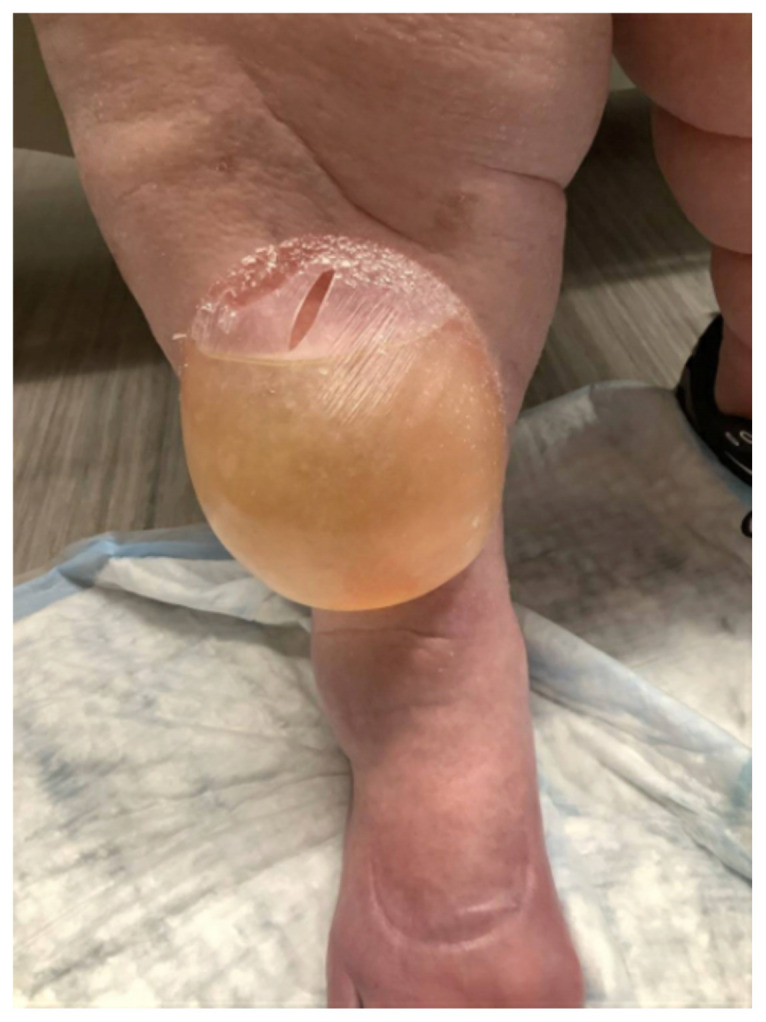


## Brief introduction

This case describes an elderly patient with congestive heart failure who presented to the emergency department (ED) with a large edema bulla on her right leg. Edema bullae are benign, non-erythematous blisters filled with serous fluid that develop in the setting of volume overload.^1^,^2^ They typically measure between one and five centimeters in diameter. The diagnosis is primarily clinical, and management focuses on treating the underlying edema which often leads to resolution of these lesions.^2^ The frequency of these bullae is difficult to gauge because there is limited published information about them. Written consent was obtained for publication of this case report.

## Presenting concerns and clinical findings

The patient is a 75-year-old female with past medical history of atrial fibrillation on apixaban, congestive heart failure on furosemide, and obesity who presented with two days of swelling to her anterior right shin after running out of her home medications. On the day prior to presentation, she noticed a “gumball” size blister on her anterior right shin. The blister rapidly expanded prompting her visit to the ED. She denied any trauma, friction injury, associated pain, fever, or infectious symptoms. Vital signs were normal. Physical exam demonstrated a right anterior shin 10 x 10 cm bullous skin lesion with serous drainage and 2+ pitting edema bilaterally. There was no surrounding erythema or elicited tenderness. Labs including a complete blood count and metabolic panel were unremarkable. Dermatology was consulted and confirmed the diagnosis of a large edema bulla.

## Significant findings

This image shows a large edema bulla on the patient’s right shin. The bulla is 10 x 10 cm, filled with serous fluid, has a spontaneously occurring defect in the skin of the superior portion of the bulla, and is non-erythematous. The bulla is much larger than the 1–5 cm edema bullae described in the literature. As edema bulla is primarily a clinical diagnosis, taking the full history and physical exam into account is essential to recognize these bullae.

## Patient course

Upon arrival in the ED, a history was obtained, physical exam performed, and basic labs were drawn. A complete blood count and metabolic panel were unremarkable. The presence of a non-erythematous bulla filled with serous fluid, in conjunction with recent lapse in diuretic therapy after the patient ran out of medication, suggested the diagnosis of edema bulla. The physical exam findings of bilateral pitting edema, indicating volume overload, along with the patient’s history of heart failure, advanced age, and limited mobility, further supported the diagnosis. However, the diagnosis was challenged because the size of this bulla was much larger than the 1–5 cm described in the literature. Dermatology was consulted, confirming the diagnosis of edema bulla. The bulla was lanced with a sterile needle and drained in the ED, and the wound was dressed. The patient was discharged with a renewed furosemide prescription and wound care instructions. These included applying petroleum jelly three times daily and vinegar compresses twice daily. On follow-up three months later, the patient reported near-complete healing of the wound, which was aided by at-home nursing care. There were no complications or adverse effects reported, and the patient adhered to the recommended treatment plan. In this case, draining the bulla followed by appropriate wound care management and treatment of the underlying problem of volume overload resolved the edema bulla.

## Discussion

This case outlines the manifestation of a large edema bulla in an elderly patient with congestive heart failure and inability to access her diuretic medication. Edema bullae are non-erythematous blisters that arise in the setting of fluid overload.^1^ These bullae tend to gradually enlarge over a span of days before rupturing spontaneously.^3^ Edema bullae are more prevalent in the elderly population, likely due to the decreased density of collagen and elastin fibers in the dermis.^1^, ^4^ Reduced mobility also contributes to the development of edema bullae due to compromised lymphatic drainage resulting from decreased muscle pump activity.^1^ Diagnosis primarily relies on clinical evaluation and patient history. Physical exam findings of fluid overload, such as dependent edema, can help support the diagnosis.^3^ In cases where the diagnosis is unclear, a skin biopsy can help rule out alternative conditions presenting with fluid-filled bullae, such as bullous pemphigoid and pemphigus vulgaris.^1^

Typical treatment entails addressing the underlying fluid overload, often resulting in spontaneous resolution of the edema bullae.^3^,^5^ Additionally, elevating the affected limb may offer symptomatic relief.^3^ There is limited literature on the acute care for larger edema bullae such as seen in our patient. Our dermatology consultant recommended puncturing the dependent portion of the bulla with a sterile needle, draining it, and leaving the bulla roof in place as it forms a natural dressing. These treatment recommendations are consistent with the management of other large bullae conditions such as bullous pemphigoid or large friction bullae.^6^,^7^ In addition, our consultant recommended dressing the drained bulla with petroleum jelly and vinegar compresses to assist in healing and prevent infection. Vinegar compresses involve soaking a clean cloth or gauze in a diluted vinegar solution (typically 2.5–5% acetic acid, as found in household vinegar), wringing out excess liquid, and applying the compress gently to the affected area for 10–15 minutes.^8^ This can be repeated once or twice daily, ensuring the skin is monitored for irritation or adverse reactions.^6^ This is a safe nonstandard treatment adjunct used for its antimicrobial properties.^8^,^9^

Clinicians should remain aware that edema bullae may present at sizes much larger than commonly reported in the literature. Early recognition of these atypical presentations can guide timely and appropriate management.

## Supplementary Information




