# Supplementary figures and images for: Effects of Volume Overload: A Case Report of an Edema Bulla
Source: J Educ Teach Emerg Med. 2026 Jan 31;11(1):V6–8. doi: 10.5070/M5.52206 (PMC12880884; doi:10.5070/M5.52206)

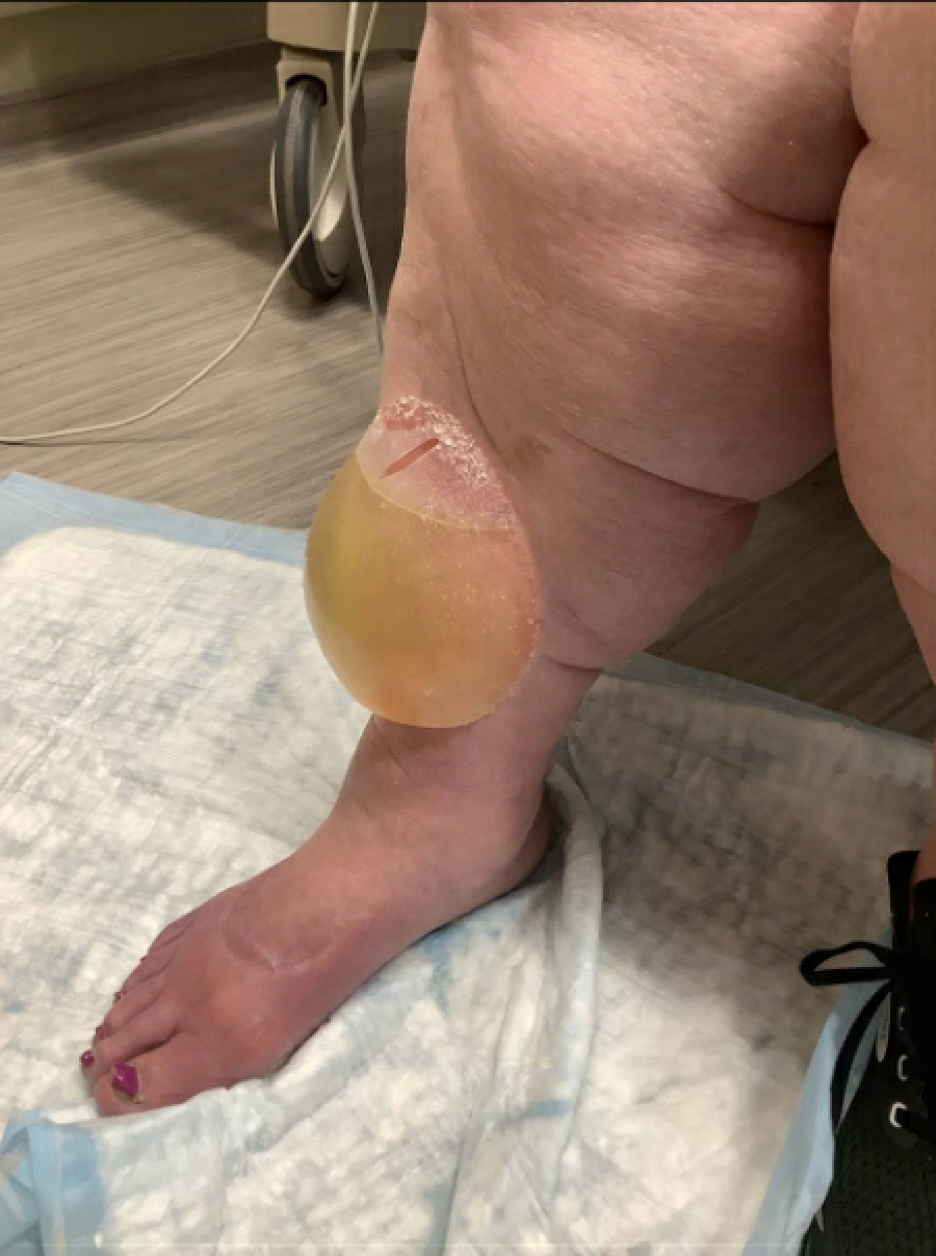

Supplement: Supplementary file 1 [file 11-1-V6-Supp1.jpeg]

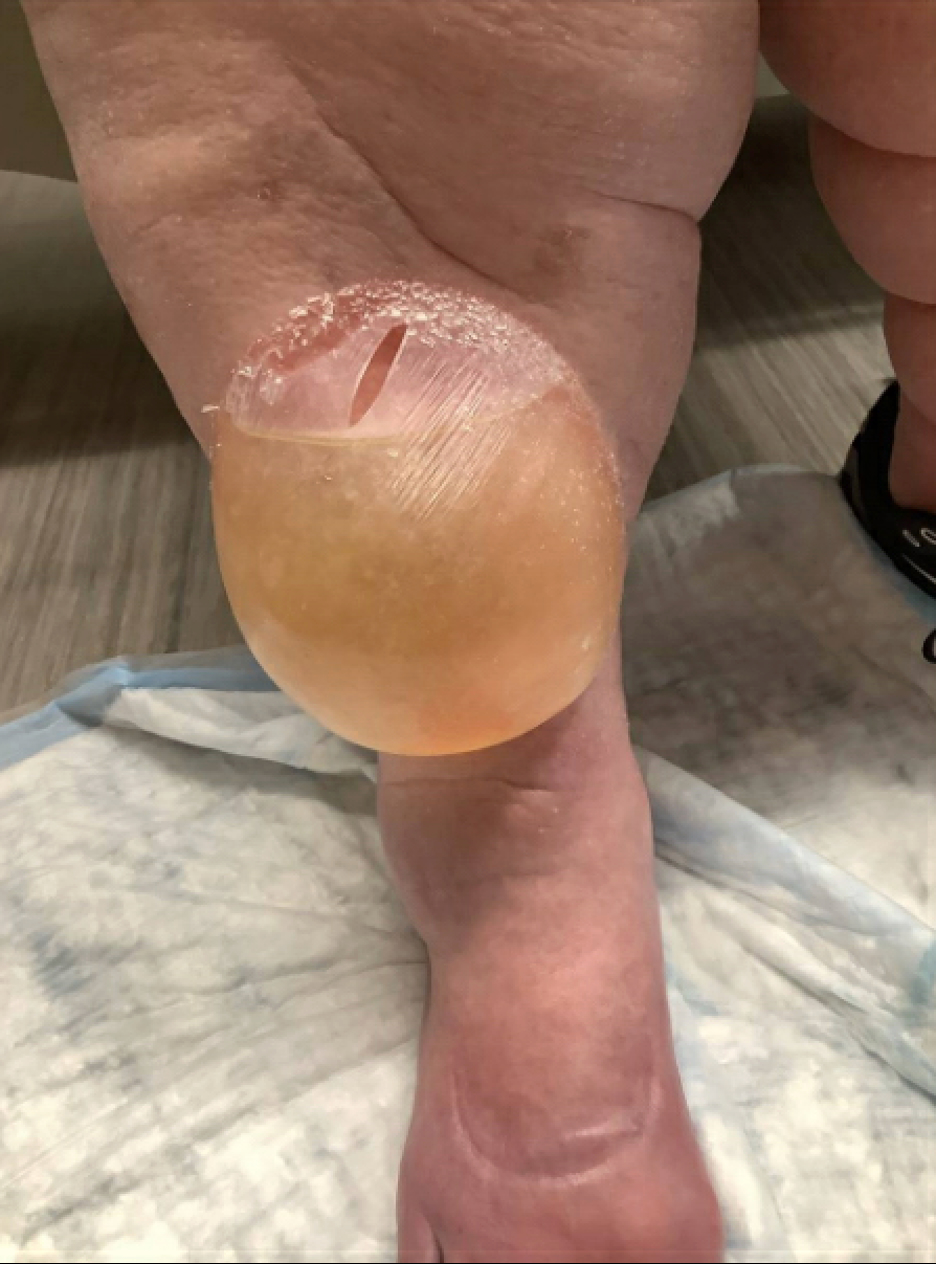

Supplement: Supplementary file 2 [file 11-1-V6-supp2.jpeg]
